# Feasibility of a reduced gadolinium dose protocol for MRI-guided radiotherapy in glioblastoma

**DOI:** 10.2340/1651-226X.2025.44022

**Published:** 2025-09-10

**Authors:** Faisal Mahmood, Uffe Bernchou, Frederik Severin Gråe Harboe, Anders Smedegaard Bertelsen, Anne Bisgaard, Rasmus Lübeck Christiansen, Bahar Celik, Elisabeth Kildegaard, Tine Schytte, Rikke Hedegaard Dahlrot

**Affiliations:** a Laboratory of Radiation Physics, Department of Oncology, Odense University Hospital, Odense, Denmark; b Department of Clinical Research, University of Southern Denmark, Odense, Denmark; c Department of Radiology, Odense University Hospital, Odense, Denmark; d Department of Oncology, Odense University Hospital, Odense, Denmark

**Keywords:** Magnetic resonance imaging, contrast media, radiotherapy, image-guided radiotherapy, glioblastoma

## Abstract

**Background and purpose:**

Magnetic resonance imaging-guided radiotherapy (MRIgRT) enables precise tumour targeting through adaptive planning, which is particularly relevant for glioblastoma due to its dynamic morphology. Gadolinium-based contrast agents (GBCAs) enhance tumour visibility, but frequent use during MRIgRT raises safety concerns related to cumulative gadolinium exposure. This study investigated the feasibility of a reduced GBCA dose protocol for patients with glioblastoma undergoing MRIgRT, aiming to balance tumour conspicuity with minimisation of GBCA-related risks.

**Patient/material and methods:**

Nine patients with glioblastoma received hypo-fractionated MRI-Linac radiotherapy (10 × 3.4 Gy) with MRI performed with either full-dose, half-dose or no GBCA enhancement. Online gross tumour volume (GTV) delineation was performed by radiation oncologists, while offline GTV delineation was independently conducted by an expert neuroradiologist on GBCA-enhanced scans. Objective assessment using automatic thresholding and a structured Likert-scale evaluation were also performed.

**Results:**

During online adaptation, GTV volumes generally remained stable or increased, whereas offline expert assessments revealed a general volume reduction and systematic volume underestimation with half-dose scans (~18%). Relative delineation volume discrepancies were most pronounced in small tumours. Structured radiologist feedback reported lower confidence, tumour conspicuity and image quality in half-dose scans, particularly for small lesions. Otsu’s thresholding revealed reduced edge definition with decreasing contrast dose. No signs of GBCA retention were observed between fractions.

**Interpretation:**

Reduced-dose GBCA-protocols are feasible. Full-dose contrast is recommended at key fractions (e.g. baseline and mid-treatment) and for small tumours, with half-dose imaging reserved for selected intervals or larger tumours. This hybrid approach may balance safety and imaging precision in adaptive MRIgRT.

## Introduction

Magnetic resonance imaging (MRI)-guided radiotherapy (MRIgRT) has emerged as an innovative approach in oncology, offering improved tumour visualisation for online treatment adaptation and precise targeting and sparing of healthy tissue [1].

Gadolinium-based contrast agents (GBCAs) play a crucial role in MRI by enhancing the visibility of tumours with high vascularity or disrupted blood–brain barriers, as well as critical anatomical structures [2]. This enhancement is vital in glioblastomas, where the tumours’ infiltrative and evolving nature presents challenges for precise imaging [3]. Moreover, glioblastomas are known to evolve during the course of radiotherapy, undergoing significant changes in size and morphology [4, 5]. These changes require frequent GBCA-enhanced imaging to adapt treatment plans effectively, ensuring accurate targeting of the tumour.

However, the frequent use of GBCAs in MRIgRT raises unresolved safety concerns. Although studies by Mahmood et al. and Wang et al. have demonstrated the stability of gadolinium chelates [6, 7], evidence suggests that gadolinium may still be retained in tissues such as the brain, bone and skin, even with stable chelates [8]. This raises concerns about potential long-term effects, particularly with repeated dosing [9].

In glioblastoma, where adaptive MRIgRT requires frequent imaging to track morphological changes, cumulative gadolinium exposure becomes a relevant safety issue. Despite the essential role of GBCAs in enhancing tumour visibility, current literature lacks specific guidelines for their use during the course of adaptive MRIgRT, especially concerning optimal contrast dose and frequency.

Addressing this gap, the present study evaluates a workflow involving the frequent use of low-dose GBCA in MRI-guided radiotherapy (MRIgRT). The aim was to assess the feasibility and efficacy of repeated reduced-dose gadolinium-enhanced imaging during MRI-Linac treatment of glioblastoma, with a focus on enhancing patient safety and optimising imaging protocols. Specifically, the study examined changes in gross tumour volume (GTV) delineation resulting from low-dose contrast use, using both subjective and objective evaluation methods. The general impact on perceived image quality and tumour conspicuity was also assessed.

## Patients/material and methods

This study is a prospective observational cohort study, reported in accordance with the STROBE (Strengthening the Reporting of Observational Studies in Epidemiology) guideline [10].

### Patients

Nine patients with glioblastoma eligible for post-operative radiotherapy were prospectively included under The Multi-OutcoME EvaluatioN of radiation Therapy Using the MR-Linac Study (MOMENTUM) [11] and treated with hypo-fractionation (10 fractions of 3.4 Gy, 5 days per week) on a 1.5 T MRI-Linac (Unity, Elekta AB, Stockholm, Sweden).

### Imaging and GBCA protocol

Patients had planning scans at a 1.5 T MRI simulator (Ingenia, Philips Medical Systems Nederland B.V., Best, the Netherlands) immobilised in the treatment position. T1W 3D fast field echo scans (TE = 3.7 ms, TR = 25 ms, FA = 30, slice thickness = 1.0 mm (no gap), in-plane pixel = 1 × 1 mm^2^, matrix = 248 × 251) were acquired both without and with intravenous administration of standard dose GBCA (Gadovist, Bayer A/S, Copenhagen, Denmark). Treatment planning system Monaco (Elekta AB, Stockholm, Sweden) was used to create the dose plan based on the GTV defined using the GBCA-enhanced scan. This plan was used as reference prior to adaptation during the first fraction at the MRI-Linac.

**Figure 1:**
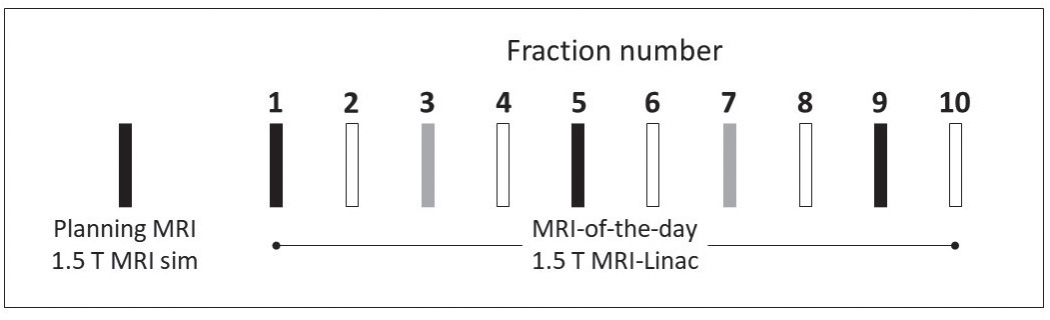
Gadolinium-based contrast agent dosage protocol for T1W-MRI during MRI-Linac treatment. Black filled = full dose, Gray = half dose, black outline = without GBCA.

In the MRI-Linac workflow, the *image-of-the-day* included one T1W 3D fast field echo scan (TE = 3.6 ms, TR = 8 ms, FA = 8, slice thickness = 1.1 mm (no gap), in-plane pixel = 1.1 × 1.1 mm^2^, matrix = 256 × 254). GBCA (Gadobutrol, Bayer A/S, Copenhagen, Denmark) was administered intravenously via a peripheral cannula (BD Nexiva^TM^) using a power injector every other fraction, either as full dose (7.5 ml of 1.0 mmol/ml) or half dose (3.7 ml of 1.0 mmol/ml), followed by 30 ml saline flush, according to the protocol outlined in Figure 1. The delay between injection and scan acquisition was comparable between full-dose and half-dose scans.

### GTV delineation (online)

The GTV was adapted at the discretion of the oncologist present at fractions where either full- or half-dose GBCA was administered (fractions 1, 3, 5, 7, 9), using the adapt-to-shape workflow [12] as standard clinical practice. In practice, a conservative adaptation approach was applied which meant that not all discernible radiological changes necessarily let to a similar change of the GTV. The new adapted plan and its structure set were used as reference for the next fraction without GBCA (fractions 2, 4, 6, 8, 10), where the GTV was transferred rigidly to the image-of-the-day without modifications. GTV to clinical target volume (CTV) margin was zero, and a planning target volume (PTV) margin of 3 mm was used.

## Expert evaluation

### GTV delineation (offline)

Upon completion of the radiotherapy course, the MRI scans acquired at the MRI-Linac with either half-dose GBCA (9 patients × 2 fractions = 18) or full-dose GBCA (9 patients × 3 fractions = 27), in total 45 MRI scans, were anonymised and presented in random order to an expert neuro-radiologist (FSGH) for GTV delineation. The radiologist had no prior knowledge of the patients or their treatment timelines. Delineations were performed over 2 days using the treatment planning system Pinnacle version 16.2.1. (Philips Medical Systems Nederland B.V., Best, the Netherlands).

A Bland–Altman analysis was performed to compare GTV volumes acquired using full-dose versus half-dose GBCA. To account for potential longitudinal tumour volume changes which may lead to a biased comparison, delineation volumes from full-dose scans were averaged across adjacent time points before comparison with half-dose scans. Specifically, the mean GTV volume from fractions 1 and 5 (full-dose) were compared with fraction 3 (half-dose); similarly fractions 5 and 9 (full-dose) were compared with fraction 7 (half-dose).

Using the above comparison strategy, the Dice Similarity Coefficient (DSC) and Mean Surface Distance (MSD) were also calculated to compare delineations on half- and full-dose GBCA images. Images were first registered using the open source tool Elastix [13].

### Structured opinion

The expert neuro-radiologist who performed the GTV delineations also filled a five-point Likert questionnaire. The questions in the questionnaire were reviewed by the radiologist beforehand to ensure their clarity and relevance, and included evaluation of the tumour boundary (Q–A), contrast between tumour and surrounding tissue (Q–B), the level of confidence in the delineation performed (Q–C) and the overall image quality (Q–D) (Table 1).

**Table 1:** Five-point Likert questionnaire.

Q–A Tumour Boundary
How well-defined are the boundaries of the contrast enhancing (CE) part of the tumour?
1 = Not at all
2 = Poorly
3 = Moderately
4 = Clearly
5 = Very clearly
Q–B Tumour-Background contrast
How would you rate the contrast between the CE part of tumour and the surrounding tissues?
1 = No contrast (tumor indistinguishable)
2 = Low contrast (difficult to differentiate)
3 = Moderate contrast
4 = High contrast
5 = Very high contrast (tumor easily distinguishable)
Q–C Confidence in delineation
How confident are you in accurately delineating the tumor based on this image?
1 = Not confident at all
2 = Slightly confident
3 = Moderately confident
4 = Confident
5 = Very confident
Q–D Overall image quality
How would you evaluate the overall image quality? (Signal-to-noise, contrast etc.)
1 = Very poor quality
2 = Poor quality
3 = Average quality
4 = Good quality
5 = Excellent quality

## Automatic GTV delineation

Additionally, to obtain an objective assessment of the enhancing tumour area, Otsu’s method [14] was applied offline for automatic thresholding. In this approach, the clinical (online) GTV delineations were expanded 2 mm iso-tropically and used as input to the algorithm. MR images were left without normalisation since Otsu’s method is invariant under linear transformations such as rescaling and shift of pixel values. In-house code developed in Matlab R2023b (The Mathworks, Inc., Natick, MA, USA) was used for the implementation.

## Results

**Figure 2:**
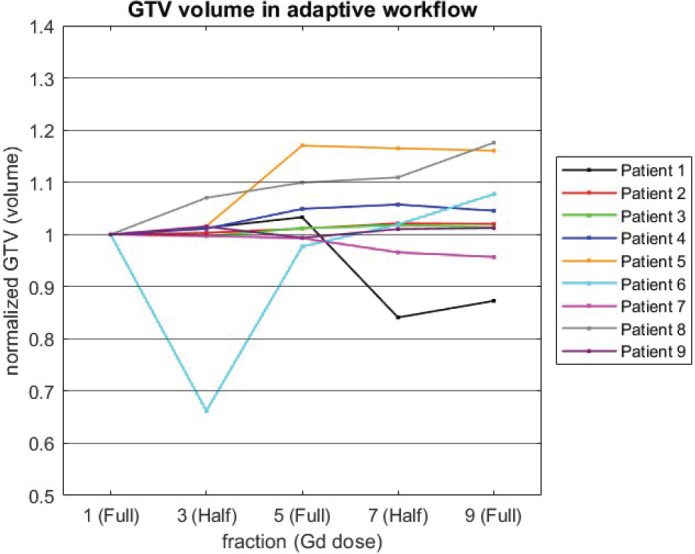
Relative volume change of the gross tumor volume adapted by the oncologist during the online adaptation workflow of the MRI-Linac.

During the online MRI-Linac treatment, the GTV adaptation performed by the oncologist led to GTV volumes remaining stable/increasing 0–10% in five patients and 10–20% in two patients, between fraction 1 and fraction 9. In two patients, GTV adaptation resulted in GTV volume decreasing 5–13% (Figure 2). Across all nine patients, the median change in GTV volume was an increase of 2%, ranging from a maximum reduction of 12% to a maximum increase of 14%.

**Figure 3:**
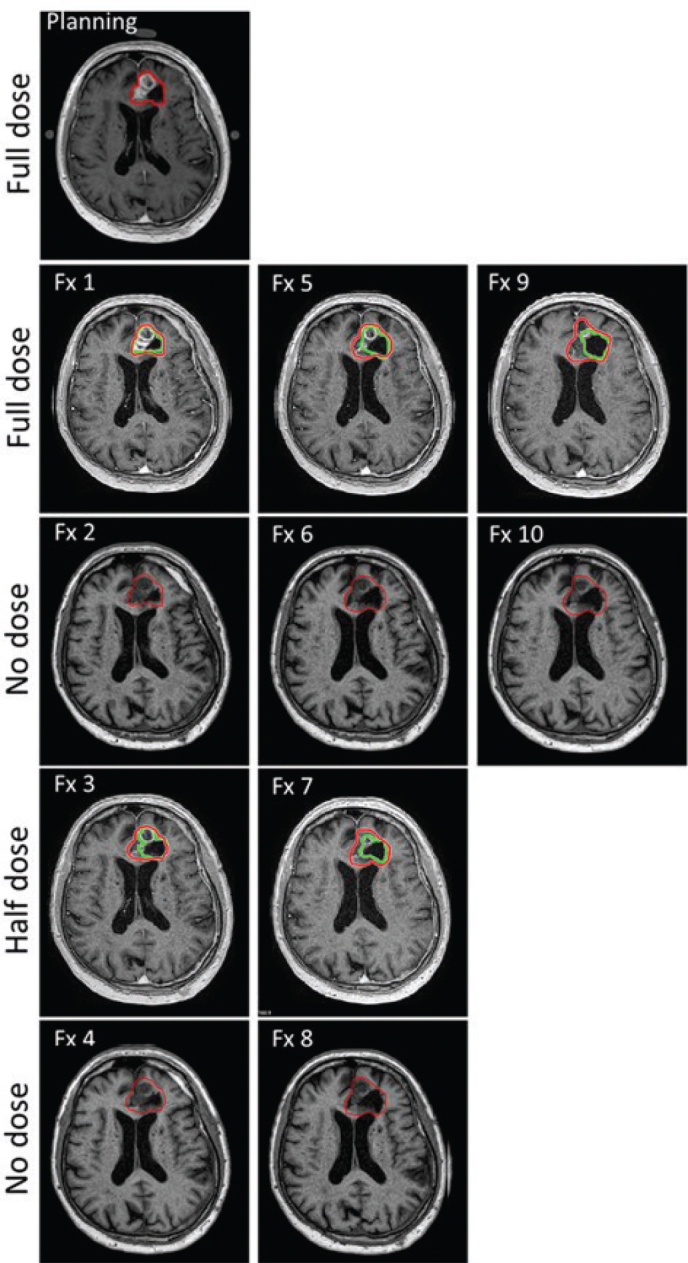
T1W magnetic resonance imaging (MRI) scans of patient 8 with decreasing tumour volume during the radiotherapy course. The online delineations (Fx 1 to Fx 10) are in red remaining relatively constant over time and the offline expert delineation are in green showing decreasing GTV volume. Fraction 1 was delivered 7 days after the planning MRI.

**Figure 4:**
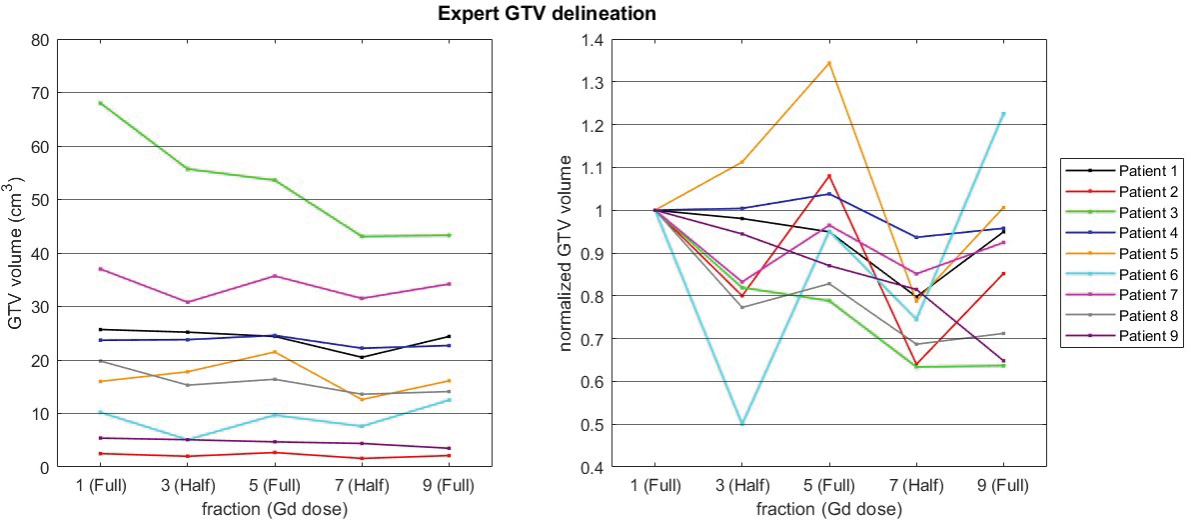
Absolute (top) and relative (bottom) gross tumor volume volumes extracted from offline expert delineations.

In comparison, the retrospective (offline) delineation of GTV performed by an expert neuro-radiologist revealed decreased/stable volume in all except one patient (patient 6). Across all nine patients, the median change in GTV volume was a reduction of 6%, ranging from a maximum reduction of 36% to a maximum increase of 22%. An example of a decreasing GTV volume as assessed by the expert radiologist is provided in Figure 3. At fractions with half-dose GBCA a dip in volume was observed for most patients in delineations performed by the expert (Figure 4, right), whereas this tendency was absent in the online clinical workflow where GTVs’ were adapted conservatively (Figure 2).

**Figure 5:**
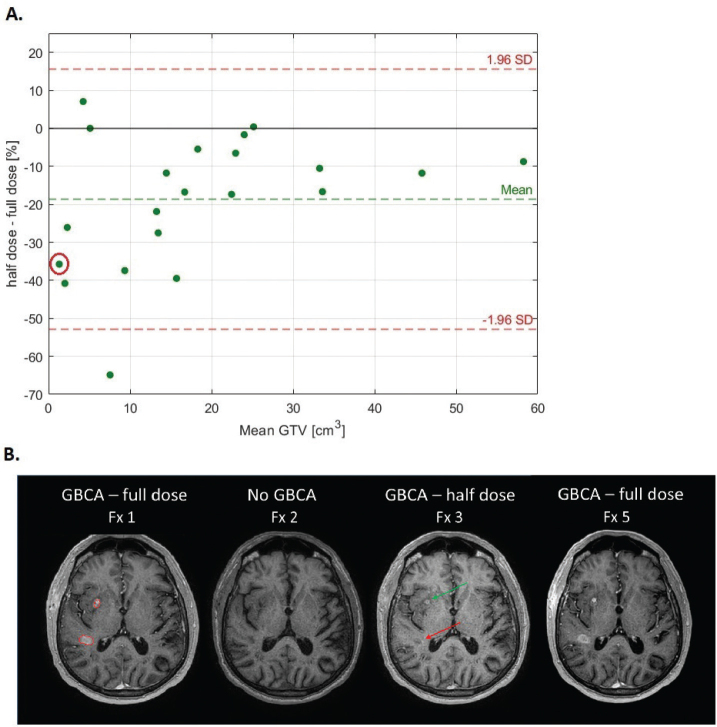
(A) The Bland–Altman plot illustrates the agreement between half-dose and full-dose Gadolinium-based contrast agent (GBCA)-enhanced magnetic resonance imaging for delineated gross tumor volume (GTV) volume. The y-axis represents the percentage difference in GTV volume between half-dose and full-dose scans, while the x-axis shows the mean GTV volume between the two methods. The limits of agreement, defined as ±1.96 standard deviations (red dashed lines), show a wide spread, with differences ranging from approximately –65% to +15%. (B) This patient had two small lesions, one of which was missed (red arrow) by the radiologist on the half-dose scan from fraction 3 due to low contrast enhancement. This was not related to actual tumour reduction, later confirmed with full-dose image at fraction 5. The second comparison with half-dose at fraction 7 is indicated with a red circle in the Bland–Altman plot.

In the smaller tumours, large relative differences were observed in delineation volume when based on half-dose versus full-dose GBCA scans (Figure 4). This observation was in agreement with the Bland–Altman analysis which indicated a systematic underestimation of GTV volume with half-dose contrast of 18% compared to the full-dose reference (Figure 5A). Furthermore, smaller tumours tend to have greater variability and larger percentage discrepancies, potentially reflecting reduced lesion conspicuity in half-dose scans, and higher sensitivity to delineation uncertainties. In one case, a small lesion was entirely missed in the half-dose scan, though it was visible in the full-dose image in later fraction (Figure 5B).

**Figure 6:**
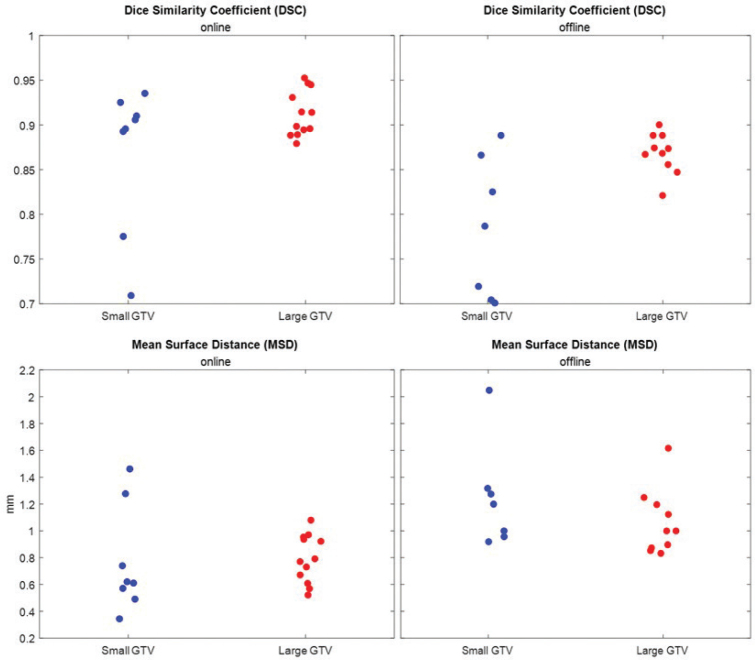
Dice Similarity Coefficient (DSC) and Mean Surface Distance (MSD) across tumours. Tumours are subdivided into small and large, using median gross tumor volume (GTV) volumes as threshold.

The DSC analyses demonstrated a generally higher DSC between half-dose and full-dose contrast in the online delineation (0.7–0.96) compared to the offline expert delineation (0.7–0.9), underlining the conservative approach of the clinical workflow (Figure 6). Likewise MSD were smaller in the online delineations (0.3–1.5 mm) compared to the offline delineations (0.8–2.0 mm). Furthermore, smaller GTVs had larger spread of DSC and MSD, and in generally performed worse.

**Figure 7:**
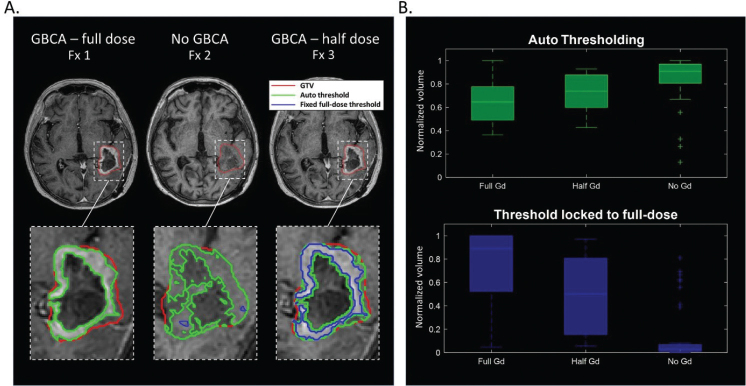
(A) Representative images of the enhancing region with three dose levels of Gadolinium-based contrast agent (GBCA). Images with less dose have reduced contrast, and less sharply defined enhancing region. Furthermore, when the threshold found in the full-dose image is applied to the images with reduced GBCA, the found enhancing regions are smaller indicating signal loss, as expected. (B) Relative volume changes of enhancing regions from all patients represented as box-plots (normalised to 2 mm gross tumor volume expansion).

Auto-thresholding using Otsu’s method revealed a less sharply defined border of the enhancing region in half-dose images compared to the full-dose images, resulting in increased volumes (Figure 7). In images acquired without using GBCA, the captured region was further enlarged and lost edge smoothness (except where it coincided with the GTV). When the full-dose threshold was applied to images with half-dose and without GBCA, the volumes reduced as expected.

There was no apparent GBCA retention from previous fractions, confirmed both visually by an expert onco-radiologist, and from the automatic thresholding.

**Figure 8:**
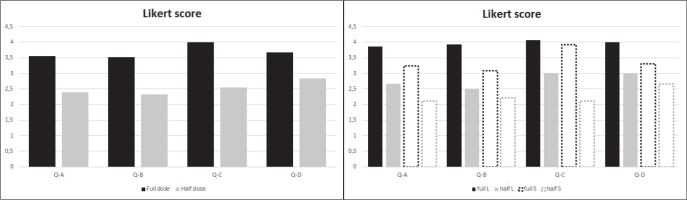
Results of the five-point likert score questionnaire. Top, the overall Likert score. Bottom, the Likert score split between small (S) and large tumour volumes (L), with cutoff point being the median volume of expert gross tumor volume delineations on all available scans. Q-A: ‘How well defined are the boundaries of the contrast enhancing (CE) part of the tumour?’, Q-B: ‘How would you rate the contrast between the CE part of tumor and the surrounding tissues?’, Q-C: ‘How confident are you in accurately delineating the tumor based on this image?’, Q-D: ‘How would you evaluate the overall image quality? (Signal-to-noise, contrast etc.)’.

The structured expert opinion showed an overall reduction in score when half-dose GBCA images were used for delineation (Figure 8, left). Average score differences ranged between 0.8 and 1.4 on the five-point Likert scale, with the largest difference related to the expert’s confidence in the delineation (Q–C), and the smallest difference in the perceived general image quality (Q–D). Splitting scores into tumour volumes below and above the median volume, two observations were made (Figure 8, right): (1) The full-dose GBCA images scored consistently higher on average regardless of tumour size. (2) A clear overall reduction of scores was observed in the small tumours compared to large tumours. An exception to this was the expert radiologist’s confidence in delineation (Q–C), which was unaffected by tumour size and scored consistently high with full-dose images. Tumour boundary definition (Q–A), tumour-background contrast (Q–B) and overall image quality (Q–D) scored clearly lower in the small tumours at both GBCA dose levels.

## Discussion and conclusion

This study was motivated by the potential risks associated with repeated administration of GBCAs, particularly in radiotherapy workflows requiring frequent imaging such as MRI-guided radiotherapy. In glioblastoma, where tumour morphology can evolve over the course of treatment, frequent contrast-enhanced imaging can be essential for maintaining precision in delineation. Our findings suggest that while reduced-dose GBCA imaging is feasible, it may compromise delineation accuracy, particularly for small lesions, supporting a cautious and individualised approach to GBCA dose reduction. Whereas it is not possible based on our data to firmly suggest a threshold, data indicate that volumes less than about 16 cm^3^ are affected relatively more than those larger (Figure 5). This was further backed up by the DSC and MSD analyses in particular. However, the clinical impact may not be correlated to this, but rather to the absolute volume missed which was not investigated.

There was no indication of GBCA retention from previous fractions, confirming the short elimination half-life of GBCAs in patients with normal kidney function (about 1.6 h) [15]. Nevertheless, subclinical retention in tissues such as brain, CSF, bone, skin, and liver has been reported [8], and repeated dosing during MRIgRT may result in cumulative exposure with potential late effects [9], supporting the need for reduced GBCA protocols.

Importantly, while the overall survival for glioblastoma patients is limited, many undergo numerous contrast-enhanced scans during radiotherapy and follow-up, resulting in non-negligible cumulative gadolinium exposure. Moreover, as treatment responses vary, some patients may experience prolonged survival, making long-term safety considerations still relevant.

Furthermore, in our study, all patients received Gadobutrol (Gadovist^®^), a macrocyclic GBCA with high kinetic stability and minimal reported tissue retention. Although gadolinium deposition has been primarily associated with linear agents, and preferentially observed in regions such as the dentate nucleus and globus pallidus, we did not perform targeted signal intensity analysis in these areas, as our MRI protocol was not optimised for detecting such changes. Given the short treatment timeframe and the contrast agent used, any deposition-related signal alterations would be unlikely in this cohort.

A strength of this study lies in the combined subjective expert evaluations with an objective, reproducible method using Otsu’s thresholding. This multi-faceted approach allowed us to capture both the perceptual impact of GBCA dose reduction on clinical decision-making and the *algorithmic* consequences. The expert radiologist’s structured opinion showed consistent reductions with half-dose imaging, especially for small tumours, underscoring the importance of full-dose imaging for clear tumour conspicuity in challenging cases. Meanwhile, the thresholding method was used as a contrast-sensitive proxy for delineation performance that captured the loss of in particular edge definition of tumour.

Limited number of studies have explored low-dose GBCA protocols in diagnostic neuroimaging, often showing that image quality can be maintained for general anatomical assessment [16]. One study reported on the feasibility of reduced GBCA dose for perfusion MRI [17]. However, these studies are not focused on radiotherapy settings where high spatial precision and consistency over time can be critical. In this context, our study extends the field by addressing not only visibility but actionable delineation in a treatment setting requiring high reproducibility and confidence.

A notable observation was the discrepancy between GTV volume changes in the online adaptation workflow and those observed in the blinded offline assessment. While clinical adaptations tended to be conservative, expert delineations revealed greater variability, particularly between full- and half-dose scans. This highlights a potential limitation of online workflows that prioritise efficiency and continuity over accurate adaptation, particularly when image conspicuity is compromised. It emphasises the need for caution when implementing GBCA dose-reduction strategies. Furthermore, in the online MRI-Linac workflow, the oncologist adapts the reference GTV from the previous fraction, which may introduce a delineation bias.

Based on our findings, a feasible GBCA protocol for MRIgRT could prioritise full-dose scans at key fractions, such as baseline, mid-treatment, and end-of-treatment, while considering half-dose imaging for interim fractions, particularly in larger tumours. For small lesions, more frequent full-dose imaging is likely warranted. This hybrid strategy could reduce cumulative gadolinium exposure while preserving the adaptive value of contrast-enhanced imaging.

Emerging alternatives may also support contrast agent dose reduction. A recently approved contrast agent with higher relaxivity offers comparable enhancement at lower doses and may be particularly suitable in adaptive workflows [18]. Additionally, artificial intelligence (AI)-based reconstruction or segmentation tools trained on full-dose datasets could be adapted to interpret low-dose scans, potentially enabling virtual dose enhancement without increasing patient exposure [19, 20]. Future work in this direction may further bridge the gap between safety and diagnostic utility.

This study has several limitations. The sample size was modest, comprising only nine patients, which limits the generalisability of our findings. Moreover, only a single expert performed the retrospective delineations, introducing potential observer bias. A larger, multi-reader study would increase robustness of the conclusions. Further, the impact of GTV delineation differences on the delivered radiation dose was not investigated, and consequently not any correlation with clinical endpoints such as local control or survival either. Finally, our study focused on a specific MRI sequence and contrast agent; results may differ with other protocols or field strengths.

In conclusion, GBCA-enhanced MRI remains essential for adaptive MRI-guided radiotherapy of glioblastoma. While half-dose imaging may be adequate for monitoring, full-dose GBCA is preferred for treatment adaptation, particularly at selected fractions. For small tumours, full-dose imaging is recommended throughout. A feasible low-dose protocol could include full-dose imaging at the first and mid-treatment fractions, with optional half-dose imaging toward the end. However, in patients with small or poorly defined tumours, more frequent full-dose scans should be considered.

## Data Availability

The data analysed in this study are subject to the following licenses/restrictions: The data are not publicly available. Informed written consent was obtained from all patients to include the data in the Momentum study (a database where data is shared between hospitals in the Elekta MRI-linac Consortium). Requests to access these datasets should be directed to The Momentum data management task force. https://mrlconsortium.org/introduction-to-momentum/.
